# Optical Skyrmions Modulation Based on Polarization Helicity

**DOI:** 10.1002/nap2.70124

**Published:** 2026-05-06

**Authors:** Dong Xu, Tingting Nie, Huajie Hu, Yujie Zhou, Xinzhong Li, Hehe Li

**Affiliations:** ^1^ Henan University of Science and Technology Luoyang China

**Keywords:** optical skyrmion, optical vortex, radially polarized light, tight focusing

## Abstract

In recent years, optical skyrmion has become a research hot spot because of its unique topological property, its flexible modulation technique is needed in the practical application fields. In this paper, by introducing the polarization helicity which characterizes the global twisted distribution of the polarization state, we investigate the spin skyrmion topological state modulation in the tight focusing of the radially polarized vortex light. We show that, the Néel‐Bloch‐Néel transition of the spin skyrmion texture can be realized in the focal plane. More interestingly, besides the stable spin Néel‐type and the Bloch‐type skyrmion can be obtained when the initial phase of incident polarization state takes some specific values, its handedness also can be well controlled. The global twisted polarization distribution plays a key role in the spin skyrmion texture modulation and the light–matter interaction. Our results provide a new idea for the dynamic modulation of spin skyrmion and have potential applications in the field of the nanophotonics and the metrology.

## Graphical abstract

The polarization helicity represents the global twisted property of the polarization state distribution, it can be used to modulate the spin skyrmion texture in the tight focusing of the radially polarized vortex light. By adjusting the polarization helicity of incident field, the Néel‐Bloch‐Néel transition of the spin skyrmion texture can be realized, and its handedness also can be well controlled.

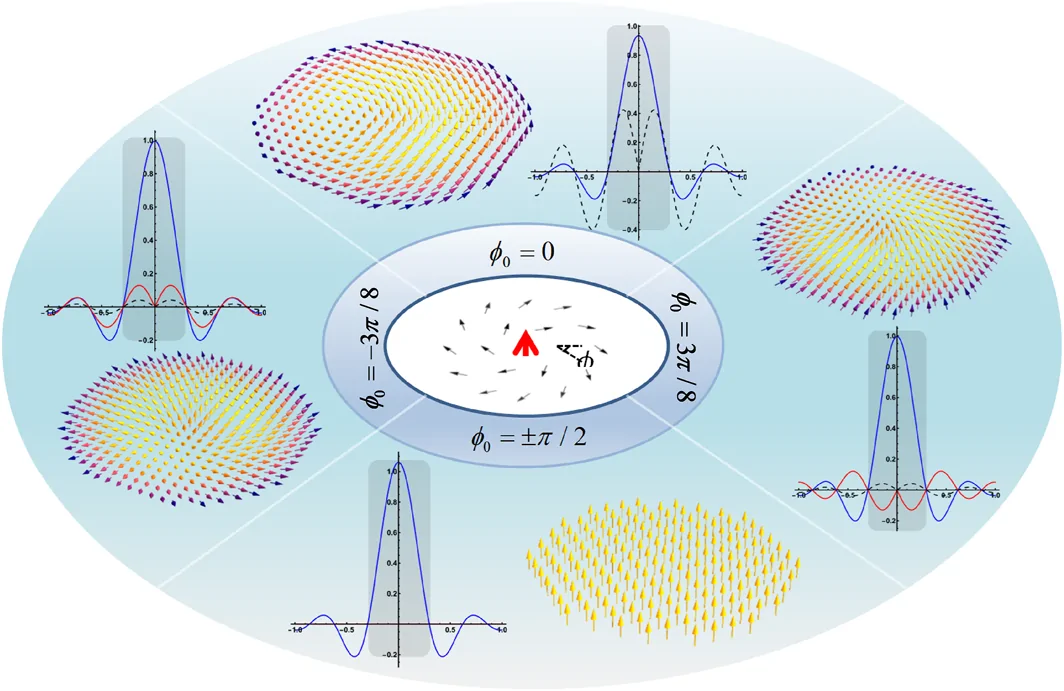

## 1 Introduction

Skyrmions indicates a topological stable vector field configurations with the quasiparticle behavior, which have been widely studied in high‐energy physics, condensed matter physics, acoustics and optics. Because of its stable structure and topological protection characteristics, skyrmions have broad applied in the fields of information processing, high‐density data storage and precision measurement [1, 2, 3].

Optical skyrmions can be formed by constructing vector structure of electromagnetic parameters in different optical systems, such as electric field component of evanescent waves [4, 5, 6, 7, 8] and free space 4π focusing system [9, 10, 11], electromagnetic vectors of super toroidal pulses [12, 13], Stokes vector of paraxial Poincaré beams [14, 15, 16, 17, 18, 19, 20], Poynting vector of counter propagating vector beams [12], optical spin angular momentum of focused vector beam [5, 21, 22, 23, 24, 25, 26], and surface plasmon polariton on metal film [27, 28, 29, 30]. According to these field vectors orientation, the topological texture of optical skyrmions is generally classified into Néel‐type, Bloch‐type, and anti‐type skyrmions. Due to its new optical characteristics in various optical systems, optical skyrmions show some potential applications in the fields of polarization sensor [21], nanopositioning [31, 32, 33, 34], super‐resolution microscopy [35, 36] etc. Then, how to construct optical skyrmions in real application scenarios using different parameters and modulate its topological texture for the practical requirements are of great significance.

Generally, the topological texture modulation of optical skyrmions can be realized by designing spatial structures on the substrate and regulating its excitation optical beams. The localized plasmon skyrmions with alternating positive and negative topological charges are observed by designing a space‐coiling meta‐structure [37]; or with the help of the spatial light modulator, the transform between various optical skyrmions [14] and its dynamic position control [21] can be achieved flexibly. Based on an optical microring cavity, the full Poincaré beam with tailored skyrmion numbers can be generated [19]. Moreover, by designing electromagnetic field textures, optical skyrmion structure based on spin or pseudospin vector can be constructed in free‐space. The flexible modulation of optical skyrmions is beneficial to extend the application ability in real scenario.

Here, we investigate the optical spin skyrmion modulation in the tight focusing of the radially polarized vortex light. We show that, the initial phase of incident polarization state leads to a twisted structure of the polarization state distribution which can be represented by the polarization helicity. We find that, the global twisted polarization state distribution will directly affect the property of the focal field, and the spin skyrmion texture is obtained in the specific domain of the focal plane. Then, by modulating the polarization helicity, the Néel‐Bloch‐Néel transition of the skyrmion texture is achieved in the focal field. Furthermore, the opposite polarization helicity can lead to the opposite handedness of the Néel‐type spin skyrmion. Our results provide a potential method for the regulation of optical spin skyrmion, and has potential applications in the field of the nanophotonics and the metrology.

## 2 Theoretical Model

Firstly, let us consider the tight focusing of a radially polarized vortex light. The analytical expression of the focal field components is derived with the help of the Richards–Wolf diffraction integral directly, and then the spin vector structure in the focal plane can be analyzed directly. The complex amplitude of incident radially polarized vortex light can be simply described in the form as ${\vec{E}}_{in}=\exp\left(il\varphi \right)\vec{u}$, l is the vortex charge, $\vec{u}={\left[c_{x}\left(\varphi \right)\;c_{y}\left(\varphi \right)\right]}^{T}$ is the basis vector of polarization state, where $c_{x}\left(\varphi \right)=\cos\left(m\varphi +\phi_{0}\right)$, $c_{y}\left(\varphi \right)=\sin\left(m\varphi +\phi_{0}\right)$, m is the polarization order, $\phi_{0}$ is initial phase of the polarization state. In fact, the basis vector of polarization state $\vec{u}$ can be rewritten in another form, $\vec{u}=R\left(\phi_{0}\right){\left[\cos\;\varphi \;\sin\;\varphi \right]}^{T}$, where

(1) $$R\left(\phi_{0}\right)=\left[\begin{array}{cc} \cos\;\phi_{0} & -\sin\;\phi_{0}\\ \sin\;\phi_{0} & \cos\;\phi_{0} \end{array}\right].$$

Obviously, $R\left(\phi_{0}\right)$ is the rotation matrix, and it indicates a rotation operation on the local basis vector of polarization state and then leads to a global twisted property of the polarization state distribution. For the high‐order radial polarization state (m>1), the initial phase $\phi_{0}$ just lead to the whole rotation of the polarization state distribution, and doesn't change its distribution property [38]. Then, in our study, we just consider the first‐order radial polarization state (m=1). In general, it is a radial polarization state when $\phi_{0}=0$, and azimuthal polarization state when $\phi_{0}=\pi /2$. Alternatively, the polarization state distribution of the radially polarized light demonstrates a macroscopical “twist” property which is well controlled by its initial phase $\phi_{0}$, as shown in Figure 1. The global twisted distribution will directly affect the spin–orbit coupling and induce the optical spin–orbit Hall effect [38, 39, 40]. To further characterize this global twist property of the polarization state distribution induced by its initial phase, a definition of the polarization helicity for the radial polarization state [39], as the following form

(2) $$h_{p}=\int_{V}\left(\nabla \times \vec{u}\right)\cdot \vec{k}dV,$$

where $\vec{k}$ is the wave vector of incident light. The curl of the polarization state is obtained directly, $\nabla \times \vec{u}=\sin\;\phi_{0}\overset{\wedge }{e_{z}}$, the polarization helicity of the radially polarized light is just determined by the initial phase of polarization state. It indicates that, the polarization helicity $h_{p}>0\;\left(0<\phi_{0}<\pi /2\right)$ and $h_{p}<0\;\left(-\pi /2<\phi_{0}<0\right)$, the corresponding polarization state distribution display the opposite twist direction. Specifically, the polarization helicity $h_{p}=0$ corresponds to the radial polarization state, and the polarization helicity $h_{p}=1$ (or −1) corresponds to the azimuthal polarization state. It knows that, the conventional optical helicity which is close related to the circular polarization state (or the vortex phase) of the light [41, 42], and are not related to the macroscopical distribution property of the polarization state. The polarization helicity represents a kind of optical chirality determined by the global twisted distribution of the polarization state determined by its initial phase, it will affect the spin–orbit coupling directly.

**FIGURE 1:**
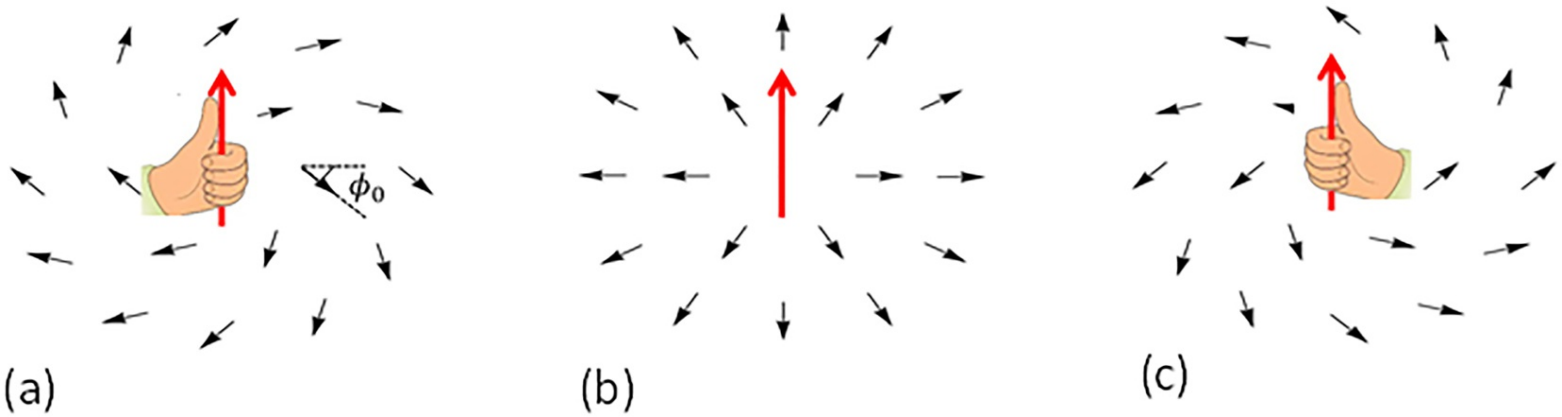
The global twisted distribution of the radial polarization state, and its corresponding polarization helicity: (a) the polarization helicity ${0<h}_{p}<1\;\left(0<\phi_{0}<\pi /2\right)$, (b) the polarization helicity $h_{p}=0\;\left(\phi_{0}=0\right)$, (c) the polarization helicity ${-1<h}_{p}<0\;\left(-\pi /2<\phi_{0}<0\right)$.

According to the Richards–Wolf diffraction integral [43] and the form of the incident light ${\vec{E}}_{\text{in}}$, the electric field components of the focal field for the tight focusing of a radially polarized vortex light are obtained analytically, as follows:

(3) $$\begin{array}{ll} E_{x}\left(r,\varphi ,z\right)= & \frac{1}{2}\pi \left\{i^{l-1}I_{+,l-1}\;\exp\left[i\left(l-1\right)\phi \right]\exp\left(-i\phi_{0}\right)+i^{l+1}I_{+,l+1}\left(t\right)\exp\left[i\left(l+1\right)\phi \right]\exp\left(i\phi_{0}\right)\right\}\\ & +\frac{1}{2}\pi \left\{i^{l+1}I_{-,l+1}\;\exp\left[i\left(l+1\right)\phi \right]\exp\left(-i\phi_{0}\right)+i^{l-1}I_{-,l-1}\;\exp\left[i\left(l-1\right)\phi \right]\exp\left(i\phi_{0}\right)\right\}, \end{array}$$

(4) $$\begin{array}{ll} E_{y}\left(r,\varphi ,z\right)= & \frac{1}{2}\pi i\left\{i^{l-1}I_{+,l-1}\;\exp\left[i\left(l-1\right)\phi \right]\exp\left(-i\phi_{0}\right)-i^{l+1}I_{+,l+1}\left(t\right)\exp\left[i\left(l+1\right)\phi \right]\exp\left(i\phi_{0}\right)\right\}\\ & -\frac{1}{2}\pi i\left\{i^{l+1}I_{-,l+1}\;\exp\left[i\left(l+1\right)\phi \right]\exp\left(-i\phi_{0}\right)-i^{l-1}I_{-,l-1}\;\exp\left[i\left(l-1\right)\phi \right]\exp\left(i\phi_{0}\right)\right\}, \end{array}$$

(5) $$E_{z}\left(r,\varphi ,z\right)=-\pi i^{l}I_{l}\;\exp\left[i\phi \right]\cos\;\phi_{0},$$

where

(6) $$I_{+,l-1}=\int_{0}^{\alpha }\left(\cos\;\theta +1\right)p\left(z\right)J_{l-1}\left(t\right)d\theta ,$$

(7) $$I_{+,l+1}=\int_{0}^{\alpha }\left(\cos\;\theta +1\right)p\left(z\right)J_{l+1}\left(t\right)d\theta ,$$

(8) $$I_{-,l+1}=\int_{0}^{\alpha }\left(\cos\;\theta -1\right)p\left(z\right)J_{l+1}\left(t\right)d\theta ,$$

(9) $$I_{-,l-1}=\int_{0}^{\alpha }\left(\cos\;\theta_{0}\theta -1\right)p\left(z\right)J_{l-1}\left(t\right)d\theta ,$$

(10) $$I_{l}=\int_{0}^{\alpha }\;\sin\;\theta p\left(z\right)J_{l}\left(t\right)d\theta .$$

Here, $p\left(z\right)=-if\sqrt{\cos\;\theta }\exp\left[ikz\;\cos\;\theta \right]\sin\;\theta /\lambda$, t=krsinθ and $J_{n}\left(\cdot \right)$ is an *n*th order Bessel function, α is the maximal angle for a given numerical aperture (NA=sinα=0.85). From Equations ((3), (4), (5)), the initial phase of incident polarization state (or the polarization helicity) directly affects the focal fields properties. Figure 2 shows the intensity evolution of the focal field components with the change of the polarization state initial phase of when the incident vortex charge equals to 1. We know that the transverse and longitudinal field components can be changed by modulating the initial phase of polarization state. Especially, when the polarization helicities are opposite and the global twisted polarization state distributions are inverted correspondingly, such as the initial phase of incident polarization state takes the opposite values, such as $\phi_{0}=-\pi /4$ and $\phi_{0}=\pi /4$, the field components demonstrate the complete opposite properties. Namely, the spin angular momentum structure in the focal field also will be modulated by the polarization helicity of incident radially polarized vortex light.

**FIGURE 2:**
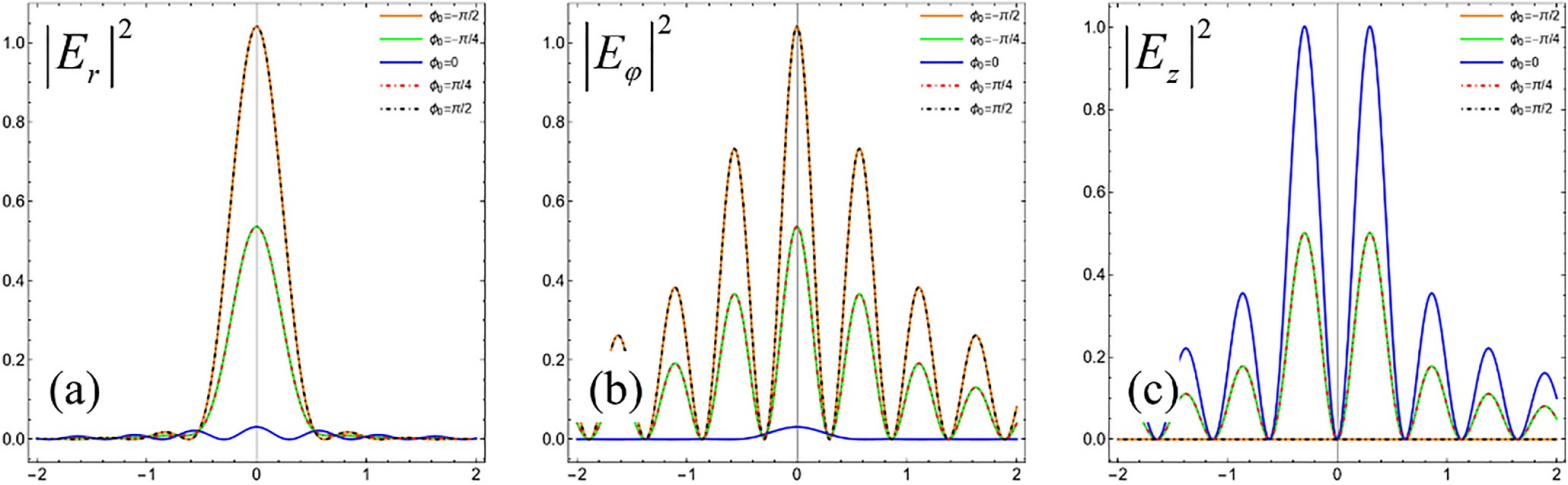
The intensity components evolution of the focal field with the different initial phase of incident polarization state when the vortex charge equals to 1.

## 3 Analyses and Discussion

According to the definition of the spin angular momentum density [44]:

(11) $$\vec{S}\propto Im\left({\vec{E}}^{*}\times \vec{E}\right),$$

the distribution property of the spin angular momentum density in the focal plane can be obtained directly. Figure 3 illustrates the spin textures properties in the focal plane when the vortex charge l=1 and the polarization helicity takes some specific values. It shows the spin vectors reverse from “up” state in the center to the “down” state at the periphery with the different skyrmion types. When the initial phase of incident polarization state takes some specific values, the polarization helicity of incident light takes some specific values, the spin vector texture demonstrates the Bloch‐type skyrmion texture or the Néel skyrmion texture in the specific region of the focal plane (the gray region in the plot of Figure 3). It shows, when $\phi_{0}=0$, the spin angular momentum vector components with $\left(0,S_{\varphi },S_{z}\right)$, the spin texture manifests the Bloch‐type skyrmion. When $\phi_{0}=\pi /4$ (or $\phi_{0}=-\pi /4$), the incident light field possesses the opposite polarization helicity, and there are three spin angular momentum vector components. In fact, the radial and the longitudinal components are dominant and the azimuthal component is small enough (almost vanishing) in the gray region in the plot of Figure 3, the corresponding purity of the local spin vector components in the focal plane [11], which is calculated by the paramater $p=\left({\left|S_{r}\right|}^{2}+{\left|S_{z}\right|}^{2}\right)/\left({\left|S_{r}\right|}^{2}+{\left|S_{\varphi }\right|}^{2}+{\left|S_{z}\right|}^{2}\right)$, remains higher than 98%. It means, when $\phi_{0}=\pi /4$ (or $\phi_{0}=-\pi /4$), because of its opposite radial components, the Néel‐type skyrmion with the opposite optical handedness is formed in the specific region of the focal plane. It indicates the chirality of the spin skyrmion in the focal plane is determined by the polarization helicity of incident light. This denotes the modulation of the skyrmion state in the focal plane can be realized by changing the polarization helicity of incident light (the global twisted distribution of incident polarization state). According to the definition of the skyrmion number [5],

(12) $$N=\frac{1}{4\pi }\iint {\vec{e}}_{S}\cdot \left(\frac{\partial {\vec{e}}_{S}}{\partial x}\times \frac{\partial {\vec{e}}_{S}}{\partial y}\right)dxdy,$$

where the vector parameter ${\vec{e}}_{S}$ can be writen as (cosα(ϕ)sinβ(r),sinα(ϕ)sinβ(r),cosα(r)), α and β are the azimuthal angle and the latitude angle of spin angular momentum vector, r and ϕ are the radial and azimuthal coordinates respectively, and it represents the unit vector in the direction of the local spin angular momentum vector. The skyrmion number indicates the variation time of the vector direction of spin angular momentum along the radial and azimuthal direction. Then, by analyzing the evolution property of spin unit vector, we know the skyrmion number is 1 when the initial phase of the polarization state $\phi_{0}=0$. Moreover, when the initial phase of the polarization state takes the other values, the skyrmion number remain unchanged and still equal to 1.

**FIGURE 3:**
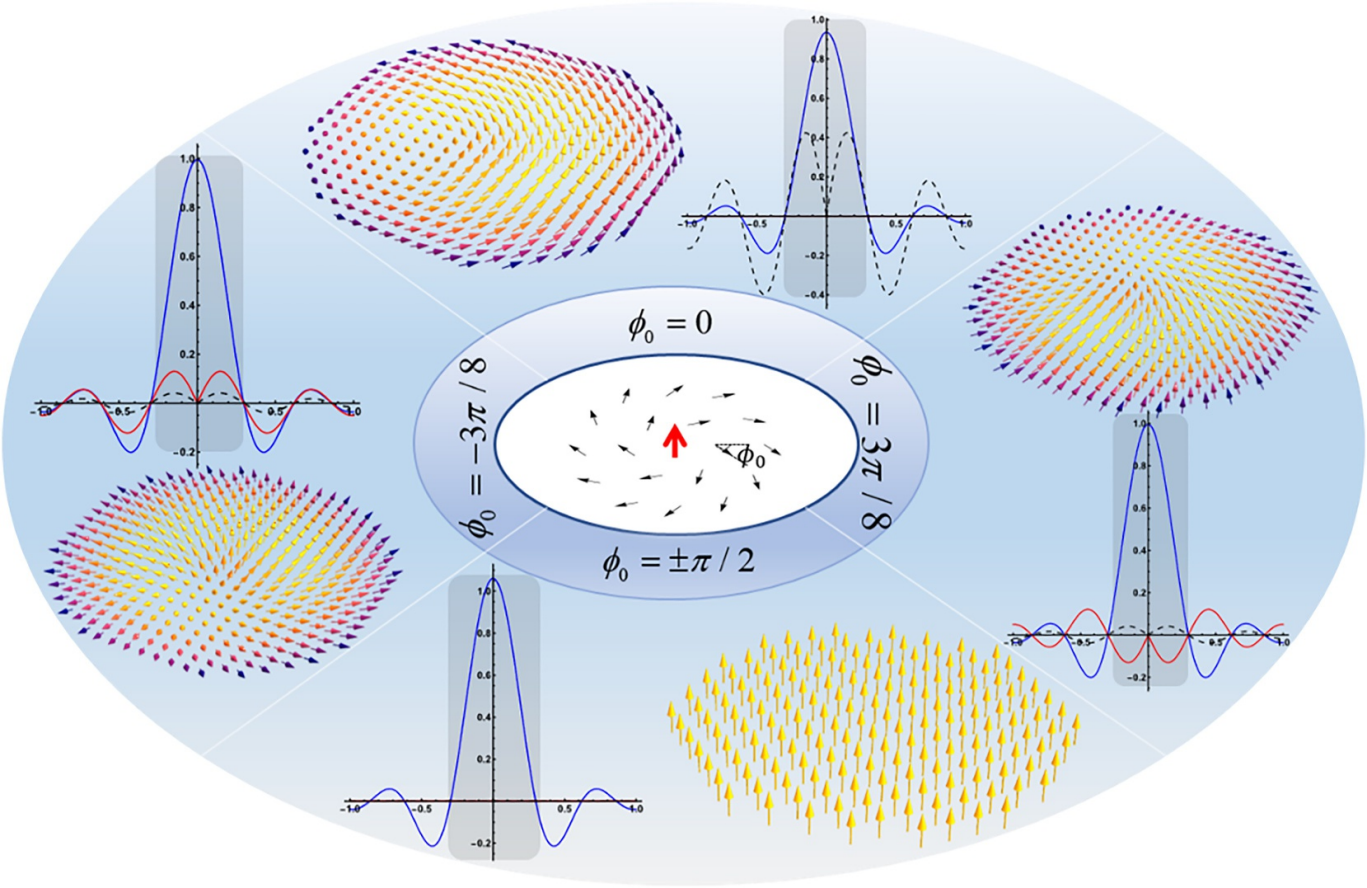
The spin textures properties in the focal plane when the vortex charge l=1 when the initial phase of incident polarization state takes some specific values, the red line, the black dotted line and the blue line in the plot indicate three components $\left(S_{r},S_{\varphi },S_{z}\right)$ of the spin angular momentum.

Figure 4 illustrates the normalized cross sections of three components of the spin skyrmion in the focal plane in detail. It shows that, the azimuthal component $S_{\varphi }$ increases gradually when the initial phase changes from $\phi_{0}=-\pi /2$ to $\phi_{0}=0$ and reaches the maximum value when $\phi_{0}=0$ and it decreases gradually when the initial phase changes from $\phi_{0}=0$ to $\phi_{0}=\pi /2$. For the radial component $S_{r}$ in the gray region in Figure 4, it is outward‐pointing ($S_{r}>0$) when the initial phase changes from $\phi_{0}=-\pi /2$ to $\phi_{0}=0$ and inward‐pointing ($S_{r}<0$) when the initial phase changes from $\phi_{0}=0$ to $\phi_{0}=\pi /2$. The interplay between the three components lead to a distribution of the orientations of the local spin vectors that is analogous to the spin skyrmion texture: when $\phi_{0}=0$, the radial component $S_{r}$ disappears, the Bloch‐type skyrmion is formed; when $\phi_{0}=\pm \pi /2$, both the radial and the azimuthal components disappear, there just is the z‐component of the spin angular momentum across the focal plane. And when the initial phase takes the other values, the spin texture displays a twisted Néel‐type skyrmion because of the nonzero azimuthal component, and its optical handedness is opposite for the different polarization helicity ($-\pi /2<\phi_{0}<0$ and $0<\phi_{0}<\pi /2$). Then, we can say the polarization helicity of incident polarization state determines the handedness of the spin skyrmions in the focal field directly.

**FIGURE 4:**
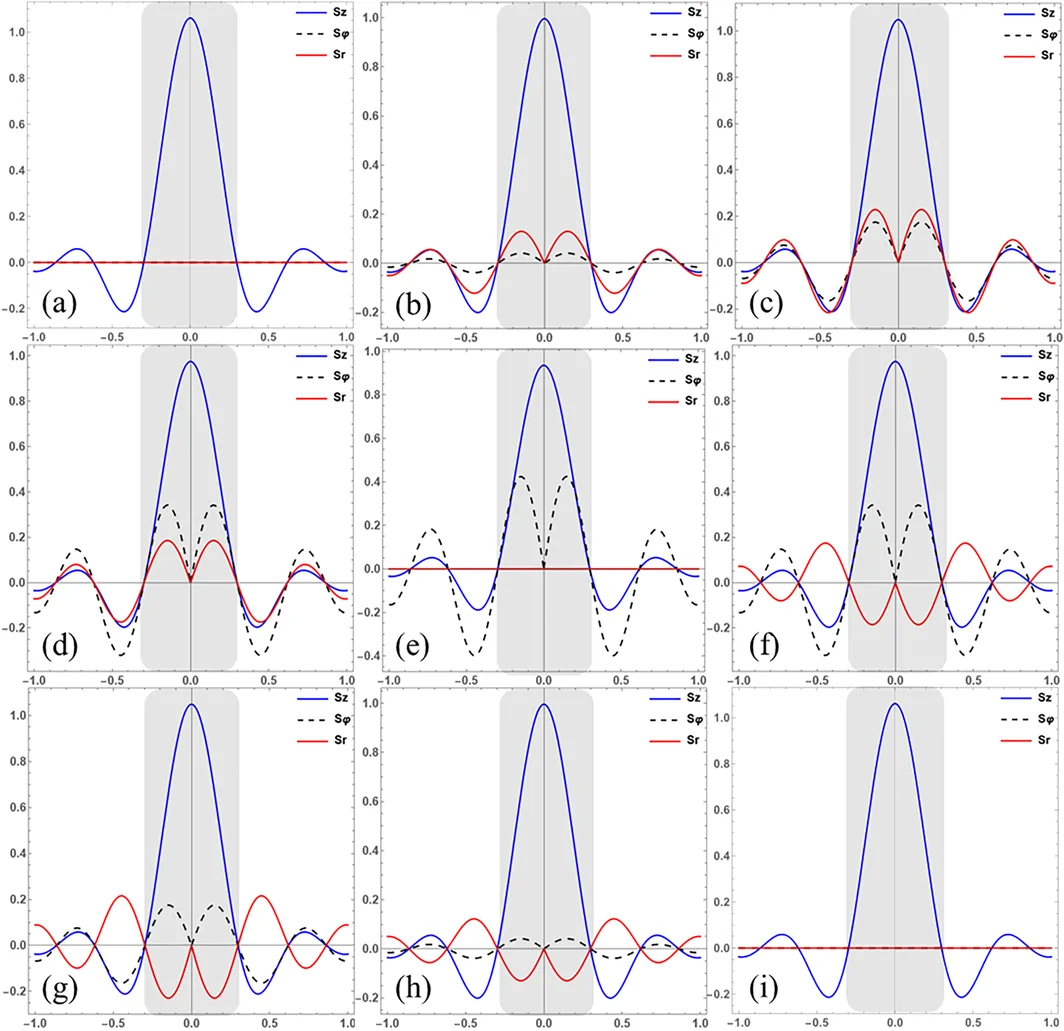
The evolution of three components $\left(S_{r},S_{\varphi },S_{z}\right)$ of the spin skyrmions in the focal plane, pictures (a) to (i) corresponds the three spin components properties when the initial phase of incident polarization state take the different values $\left(\phi_{0}=-\pi /2,-3\pi /8,-\pi /4,-\pi /8,0,\pi /8,\pi /4,3\pi /8,\pi /2\right)$, respectively.

According to the results shown in Figure 4, especially when $\phi_{0}=\pm 3\pi /8$, the corresponding purity of the local spin vector components in the focal plane can be obtained by calculating the parameter $\left({\left|S_{r}\right|}^{2}+{\left|S_{z}\right|}^{2}\right)/\left({\left|S_{r}\right|}^{2}+{\left|S_{\varphi }\right|}^{2}+{\left|S_{z}\right|}^{2}\right)$ which reach as high as 98% when $\phi_{0}=\pm 3\pi /8$, then the azimuthal component of the spin can be ignored, the spin texture can be considered as the Néel‐type skyrmion. The spin skyrmion texture is obtained in the specific region (the gray region) of the focal plane, and with the change of the polarization helicity (the initial phase of incident polarization state $\phi_{0}$ from −π/2 to −π/2) its form evolves from the Néel‐type skyrmion to the Bloch‐type skyrmion to the anti‐Néel‐type skyrmion. These results indicate the Néel‐Bloch‐anti‐Néel transition of the spin skyrmion texture can be realized by changing the polarization helicity of incident polarization state.

## 4 Conclusion

In summary, we have theoretically investigated the optical spin skyrmion modulation in the tight focusing of the radially polarized vortex light. We showed that, the global twisted distribution of the incident polarization state determined by its initial phase can be characterized as the polarization helicity, and it will directly affect the spin skyrmion texture in the focal plane. It is worth noting that the polarization helicity is just valid for the first‐order radial polarization state (m=1), this is because the rotation operation on the local basis vector of polarization state is invalid for the high‐order radial polarization state (m>1) [38]. By modulating the polarization helicity of incident light, the Néel‐Bloch‐anti‐Néel transition of the skyrmion texture can be realized in the focal plane. Meanwhile, because the sign of the polarization helicity of incident polarization state affects the handedness of the Néel‐type skyrmion in the focal field directly, then it can be said that the polarization helicity (the global twisted distribution) determines the chirality of the spin skyrmion in the focal field. These results prove that macroscopical and global distribution of optical parameters can be considered as a new modulation index for the light–matter interaction, besides the intrinsic parameters (phase, polarization state etc.). Our study provides a direct modulation technique for the spin skyrmion in the optical tight focusing and has potential applications in the field of the nanophotonics and the metrology.

## Author Contributions

**Dong Xu:** visualization, data curation, writing – original draft. **Tingting Nie:** Data curation, visualization. **Huajie Hu:** funding acquisition. **Yujie Zhou:** funding acquisition. **Xinzhong Li:** project administration, writing – review and editing. **Hehe Li:** writing – review and editing, funding acquisition, project administration.

## Funding

This work was supported by the National Natural Science Foundation of China (12474291 and 12504345), and the Natural Science Foundation of Henan Province (262300421306 and 262300420321).

## Conflicts of Interest

The authors declare no conflicts of interest.

## Data Availability

Data sharing not applicable to this article as no datasets were generated or analyzed during the current study.
